# ‘Plasma first’ approach for detecting epidermal growth factor receptor mutation in advanced non-small cell lung carcinoma

**DOI:** 10.1007/s00432-024-05828-w

**Published:** 2024-07-27

**Authors:** Amber Rathor, Prabhat Singh Malik, Pranay Tanwar, Sachin Khurana, Hemavathi Baskarane, Deepam Pushpam, Aruna Nambirajan, Deepali Jain

**Affiliations:** 1 Department of Pathology, All India Institute of Medical Sciences, New Delhi, 110029 India; 2 Department of Medical Oncology, Dr.B.R.A.IRCH, All India Institute of Medical Sciences, New Delhi, India; 3 Department of Laboratory Oncology, Dr.B.R.A.IRCH, All India Institute of Medical Sciences, New Delhi, India

**Keywords:** NSCLC, EGFR, Liquid biopsy, Plasma first, Turnaround time

## Abstract

**Introduction:**

The treatment approach for recently diagnosed advanced non-small cell lung cancer (NSCLC) with *EGFR* mutations primarily relies on confirming the tissue diagnosis as non-squamous NSCLC. This routine clinical practice of tissue diagnosis imposes several barriers and delays in turnaround time (TAT) for biomarker testing, significantly delaying the time to treatment. The objective of this study is to investigate the ‘plasma first’ approach for detection of *EGFR* mutation in advanced stage treatment naïve NSCLC patients.

**Methods:**

We prospectively collected blood samples of treatment naïve patients with clinical and radiological suspicion of advanced stage NSCLC prior to obtaining tissue biopsy. Plasma cfDNA was tested for *EGFR* mutation using two different methods. We compared the sensitivity and TAT of liquid biopsy with tissue biopsy.

**Results:**

In total, we analyzed plasma cell-free DNA (cfDNA) of 236 patients suspected of having advanced NSCLC for *EGFR* mutations. We observed a notably shorter turnaround time (TAT) of 3 days, which was significantly quicker compared to the 12-day TAT for tissue biopsy (*p* < 0.05). The ddPCR method had a sensitivity of 82.8%, which was higher than 66.34% sensitivity of ARMS-PCR. The current study also highlights that there is no significant difference in the clinical outcome of the patients whether treated based on liquid biopsy only or tissue biopsy (median progression-free survival of 11.56 vs. 11.9 months; *p* = 0.94).

**Conclusions:**

Utilizing a ‘plasma first’ strategy, given its shorter turnaround time, strong positive concordance and comparable outcomes to tissue biopsy, emerges as a highly specific and reliable method for detecting *EGFR* mutations in advanced-stage NSCLC.

**Supplementary Information:**

The online version contains supplementary material available at 10.1007/s00432-024-05828-w.

## Introduction

Lung cancer is the primary cause of cancer deaths globally, often diagnosed at an advanced inoperable stage (Hung et al. 2019). Recent treatment advancements have shifted from the traditional chemotherapeutic approach to personalized targeted approaches (Chan and Hughes 2015; Jones and Baldwin 2018) based on identifying specific driver mutations like epidermal growth factor receptor (*EGFR*) and fusions in anaplastic lymphoma kinase (*ALK*) and ROS proto-oncogene 1 (*ROS1*) (Maemondo et al. 2010; Chuang and Neal 2015). The field of precision oncology revolves around the comprehensive molecular characterization of the most common adenocarcinoma subtype of non-small cell lung cancer (NSCLC). *EGFR* and its downstream signalling pathways have been the most extensively studied key player in the tumor development of NSCLC (Chan and Hughes 2015). *EGFR* mutations are more prevalent in Asia than other geographical regions and have been reported in up to 49.1% of Asian NSCLC patients with advanced stage (Benbrahim et al. 2018; Melosky et al. 2022). Studies have reported the occurrence of *EGFR* mutations in the Indian population ranging from 23 to 44% (Sahoo et al. 2011; Chougule et al. 2013; Singh et al. 2020). Tumor acquisition is vital and testing time for drivers is the current standard for the selection of treatment in *EGFR*-mutation positive advanced stage NSCLC (Lindeman et al. 2018; Panchard et al. 2018; Singh et al. 2022). However, difficulty in acquiring tumor tissue, delay in diagnosis, inadequate tumor tissue available for molecular testing and rapid deterioration of patient’s general condition hinders timely molecular testing and early initiation of therapy (Pisapia et al. 2019).

Liquid biopsy provides a minimally invasive alternative for genotyping, overcoming limitations of conventional biopsies (Diaz and Bardelli 2014). Detecting targetable mutations from circulating tumor DNA (ctDNA), a component of circulating cell-free DNA (cfDNA) has opened up new possibilities in therapeutic decision making, offering the choice between the ‘tissue first’ versus ‘plasma first’ approach (Rolfo et al. 2021). In advanced NSCLC, ctDNA detection has been limited to patients who have either progressed on EGFR TKIs or have inadequate tumor tissue for molecular analysis (Canale et al. 2019). Liquid biopsy holds great potential for rapid diagnosis, prognosis and predicting treatment response (Kawahara et al. 2015). *EGFR* mutations are usually detected from tumor DNA in the form of formalin-fixed paraffin-embedded (FFPE) diagnostic blocks or ctDNA from plasma. The standard clinically applicable method of *EGFR* detection in FFPE is polymerase chain reaction (real-time PCR-based), while various methods have been developed for liquid biopsy samples. Next-generation sequencing based (NGS) approaches have significantly outperformed other methods with greater sensitivity but requires sophisticated computational methods and bioinformatic expertise (Lee et al. 2020). Real-time PCR-based and droplet digital PCR (ddPCR) based methods are the two most feasible clinically applicable methods that can be applied as a quick screening test for liquid biopsy samples for tumor genotyping. This strategy is more pragmatic in ethnic populations like Asians where the frequency of *EGFR* mutation is high. Hence, the aim of the present study is to evaluate the ‘plasma first’ approach using liquid biopsy in advanced stage treatment naïve NSCLC patients for early detection of *EGFR* mutation and compare it with ‘tissue first’ approach.

## Subjects and methods

### Sample collection and processing

This prospective study was approved by institute ethics committee (IECPG-740/23-12-2021) and all patients given written informed consent for blood collection. All patients enrolled (*n* = 285) in the study were presented to lung cancer clinic of the institute with clinical and radiological suspicion of lung cancer. 10mL of peripheral blood sample was collected aseptically in K2-EDTA vial prior to obtaining tissue biopsy.

Peripheral blood drawn was gently mixed by inverting the vial several times immediately and processed for plasma separation within 1 h of collection. To obtain plasma, the collected whole blood sample was centrifuged in an optimized two-step centrifugation process and stored at -80 °C until processed further for cfDNA extraction. 4mL of stored plasma was processed to isolate cfDNA using the Maxwell® RSC ccfDNA Plasma kit (Promega, USA) as per slight modifications in the manufacturer’s instructions. DNA isolated was quantified using Nanophotometer (Implen N60, US) and Quantus Fluorometer (Promega, USA) using QuantiFluor® dsDNA kit.

### Detection of EGFR mutations using ARMS-PCR

Real-time polymerase chain reaction (ARMS-PCR) was performed to detect clinically relevant *EGFR* hotspot mutations using EGFR RGQ PCR IVD kit (Qiagen, Manchester, UK). This is a ready to use kit which can qualitatively detect 29 clinically relevant hotspot mutations in the exon 18, 19, 20 and 21 of the *EGFR* gene. The qPCR assay was performed as per manufacturer’s instructions and the final interpretation of data was done according to recommended kit guidelines.

### Detection of EGFR hotspot mutations using droplet digital PCR (ddPCR)

All droplet digital PCR (ddPCR) consumables including droplet PCR supermix, droplet generation oil for probes, droplet generator cartridges and gaskets, droplet reader oil and ddPCR 96-well plates were procured from Bio-Rad Laboratories Inc. (Hercules, CA, USA). ddPCR was performed with three commercially available PrimePCR™ ddPCR™ Mutation Detection Assay Kit for E746_A750del, L858R and T790M (Bio-Rad; Hercules, CA). The PCR reaction was performed according to the manufacturers’ instructions and the droplets were read by Bio-Rad QX200 ddPCR droplet reader system and finally analysed using Quantasoft version 1.7.

### Statistical analysis

Data analysis was performed using Stata statistics version 14.2 (StataCorp LLC, USA). The Chi-Square test/Fisher Exact test was used to analyse the baseline categorical variables. Percentage of concordance between both techniques was determined from matched *EGFR* positive patients only either by tissue or liquid biopsy. We estimated turnaround time (TAT) as the time defined between the registration of the samples (tissue or liquid biopsy) in the pathology or molecular biology laboratory and the *EGFR* molecular test performed. Progression free survival (PFS) was studied using Kaplan-Meier curves, defined as the period of time from the start of TKI therapy until disease progression or death of the patient from any reason.

## Results

### Patient selection

This was a prospective study with 285 newly diagnosed/suspected treatment naïve patients. Patients’ peripheral blood was collected and plasma cfDNA isolated was quantified and checked for quality by using both spectrophotometer and fluorometer. All patients tested by liquid biopsy were diagnosed at an advanced metastatic stage of the disease with involvement of bone and brain (58.6%) as the most common metastatic sites followed by concurrent effusions and other sites such as liver, adrenal and pancreas.

### Liquid biopsy for *EGFR* molecular testing using ARMS-PCR

**Fig. 1:**
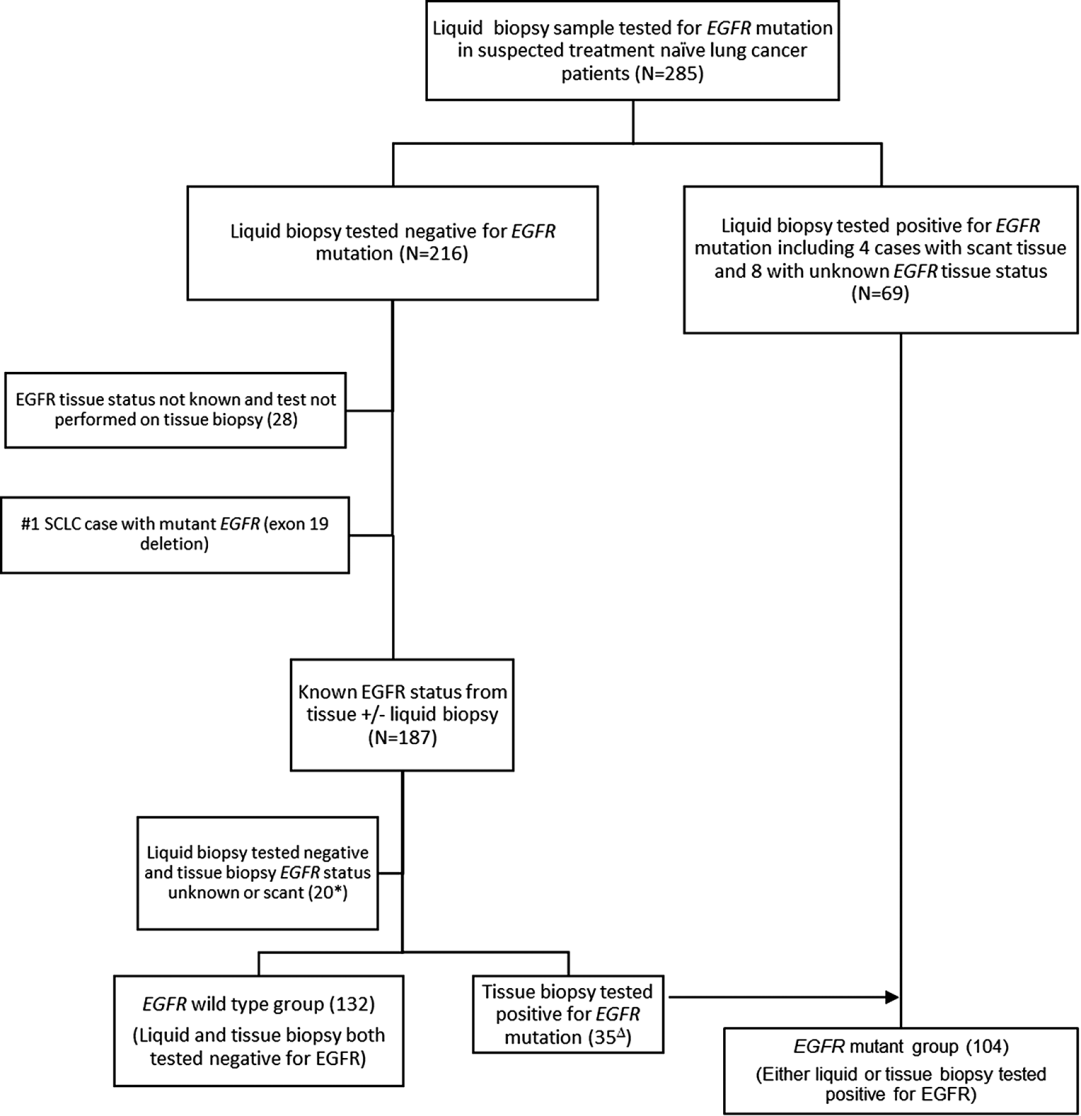
Summary of samples collected for final analysis (*excluding cases in which tissue biopsy was scant for molecular testing; Δ patients tested negative on liquid biopsy by ARMS-PCR but tested positive on tissue biopsy; #excluded a case of small cell lung cancer tested positive for EGFR mutation by liquid biopsy)

**Fig. 2:**
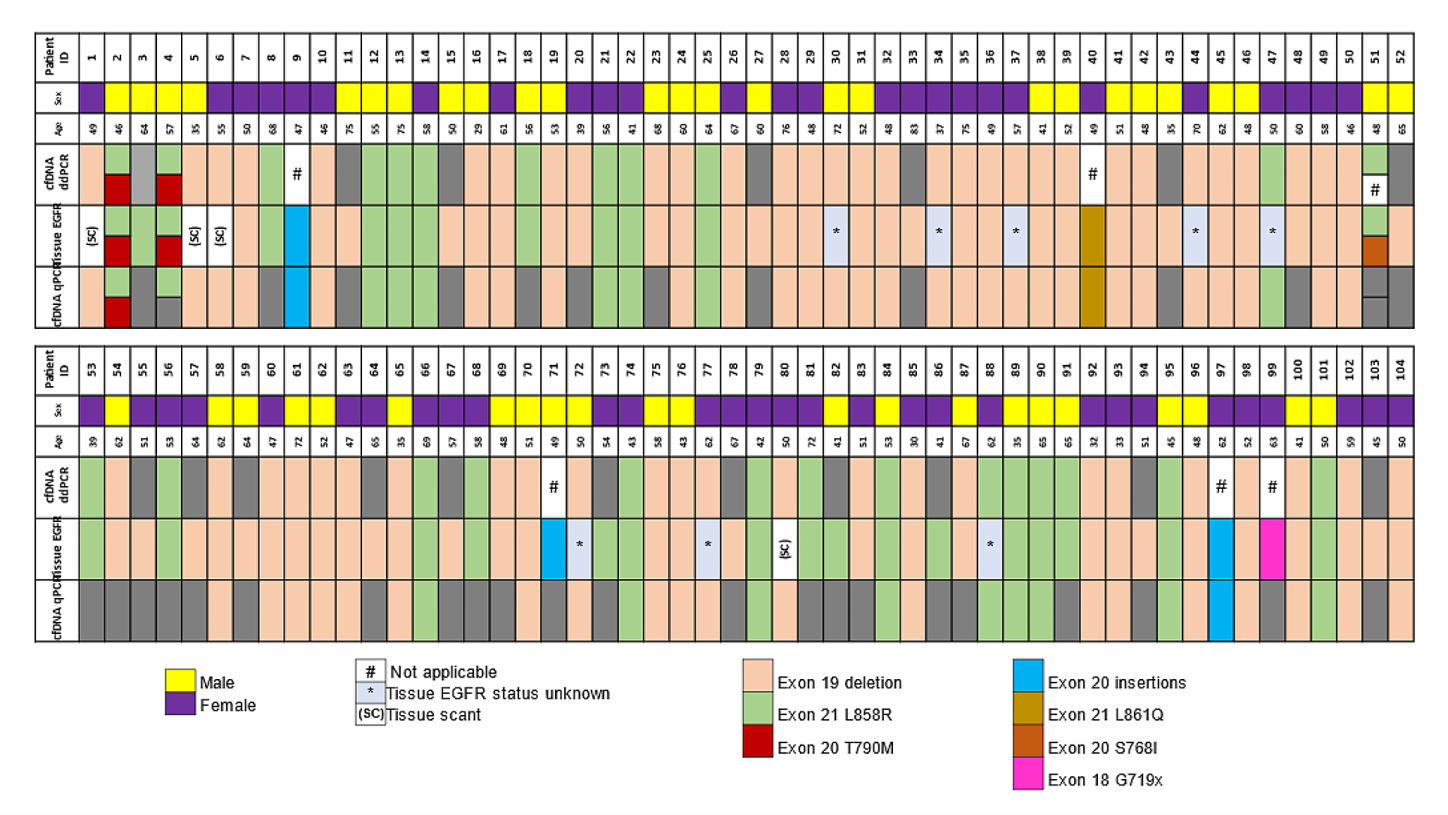
*EGFR*-mutated patients’ cohort and concordance of matched plasma cfDNA with tissue *EGFR* status as compared by ARMS-PCR and ddPCR

All patients enrolled in the study were tested for *EGFR* mutation on liquid biopsy using ARMS-PCR prior to obtaining tissue biopsy (Fig. 1). Sixty-nine (24.2%) of total 285 treatment naïve patients with suspected NSCLC were found to be positive for *EGFR* mutation by liquid biopsy only using ARMS-PCR. In these 69 cases, 48 (69.5%) showed exon 19 deletions followed by 17 (24.6%) cases of exon 21 L858R mutation. Among the ten plasma only positive patients with exon 19 deletions, four samples had insufficient tissue for molecular analysis, and the tissue *EGFR* status was unknown for the remaining six cases. Similarly, for the cases where L858R mutation was detected in plasma, the tissue status was not known for two cases. There were a few uncommon mutations such as exon 20 insertions (2), exon 21 L861Q (1) and 1 of compound mutation (exon 21 L858R and exon 20 T790M) (Fig. 2; Table 1). The median turnaround time (TAT) for detection of *EGFR* mutation using liquid biopsy was found to be 3 days (range 1 to 12 days).

### Tissue sampling and *EGFR* molecular testing by ARMS-PCR

All 285 patients enrolled in the study were followed in the clinic for availability of matched tissue biopsy and confirmed *EGFR* mutation status. Twenty-eight (9.8%) cases were excluded because of non-availability of matched tissue biopsy, inconclusive biopsy, cancer of primary origin other than lung or patient lost to follow up after diagnosis without taking treatment. One case was additionally excluded diagnosed as small cell lung cancer on tissue biopsy which however detected to have *EGFR* mutation (exon 19 deletion) on liquid biopsy testing and then confirmed on tissue biopsy. Out of the remaining 256 patients, 187 (73%) tested negative for *EGFR* mutation using liquid biopsy. Among these, 132 (70.5%) cases were found to be *EGFR* wild type when tested using both tissue and liquid biopsy (Fig. 1). Twenty (10.6%) of 187 cases tested negative for *EGFR* mutation using liquid biopsy but their matched tissue biopsy *EGFR* status was scant for molecular testing. However, 35 (18.7%) of 187 were found to be positive for *EGFR* mutation on tissue biopsy using ARMS-PCR and plasma cfDNA of these cases was further tested for *EGFR* mutation using droplet digital PCR (ddPCR).

Overall, 236 treatment naïve patients tested for *EGFR* mutation and subdivided into two groups, 104 (44.06%) patients with *EGFR* mutations and 132 (55.94%) patients with wild-type *EGFR* (Fig. 1). Patients tested for *EGFR* mutation using tissue biopsy by ARMS-PCR showed exon 19 deletions in 59/104 (56.7%), exon 21 L858R in 25/104 (24%) and uncommon mutations in 5 (3 of exon 20 insertions, 1 of exon 18 G719x and 1 of exon 21 L861Q). Additionally, 3 cases showed compound mutation of which 2 had exon 21 L858R and exon 20 T790M while 1 had exon 21 L858R and exon 20 S768I. Of these 104 cases, tissue biopsy in 12 (11.5%) was either scant or not known for *EGFR* molecular testing but their matched liquid biopsy tested positive either using ARMS-PCR or ddPCR (Table 1). The median turnaround time (TAT) for detection of *EGFR* mutation using tissue biopsy was found to be 12 days (range 7 to 57 days).

**Table 1:** Frequency distribution of *EGFR* mutation subtype as detected by tissue biopsy (ARMS-PCR only) or liquid biopsy (ARMS-PCR and ddPCR both)

Cases with EGFR mutation subtype	Tissue biopsy (ARMS-PCR)	Liquid biopsy (ARMS-PCR)	Liquid biopsy (ddPCR)
Common mutations			
Exon 19 deletions (69)	59^+4,#6^	48	55
Exon 21 L858R (27)	25^#2^	17	24
**Uncommon mutations**			
Exon 20 insertions (3)	3	2	**
Exon 18 G719 × (1)	1	0	**
Exon 21 L861Q (1)	1	1	**
**Compound Mutations**			
Exon 21 L858R & Exon 21 T790M (2)	2	2^$^	2
Exon 21 L858R & Exon 20 S768I (1)	1	0	1^Δ^

+ tissue scant for molecular analysis, # tissue EGFR status unknown, ** technically not applicable, $ L858R detected by both ARMS-PCR and ddPCR but T790M was detected by ddpcr only, ^Δ^L858R was detected using ddPCR and exon 20 S768I was technically not applicable using ddPCR

### Liquid biopsy for *EGFR* molecular testing using ddPCR

All 104 patients tested positive for *EGFR* mutations either using liquid or tissue biopsy had their plasma cfDNA tested for *EGFR* common mutations only using ddPCR except five cases with uncommon *EGFR *mutations. Using ddPCR, 55/99 (55.5%) cases showed exon 19 deletions followed by 24/99 (24.2%) of exon 21 L858R and 3 cases of compound mutation. Among the plasma negative cases, ddPCR identified 17 out of 33 false negative cases that were undetected by the less sensitive ARMS-PCR technique. However, two false negative cases, one with exon 20 insertions and one with exon 18 G719x could not be technically assessed using ddPCR. Detailed representation of matched tissue-plasma samples detected using ddPCR has been depicted in Fig. 2 and Supplementary Fig. 1.

### Concordance and turnaround time (TAT) of tissue vs. plasma cfDNA *EGFR* mutation status

Matched plasma cfDNA from 104 *EGFR* mutant group when primarily tested using ARMS PCR, 69/104 (66.34%) cases positively correlated with tissue *EGFR* status. All 69 patients’ plasma samples that tested positive for *EGFR* mutations with ARMS-PCR also showed positive results with ddPCR, except for mutations that were technically challenging to detect. We performed ddPCR further for common *EGFR* mutations and the assay sensitivity increased to 82.8% (82 out of 99 cases) when compared with ARMS-PCR (Fig. 2).

**Fig. 3:**
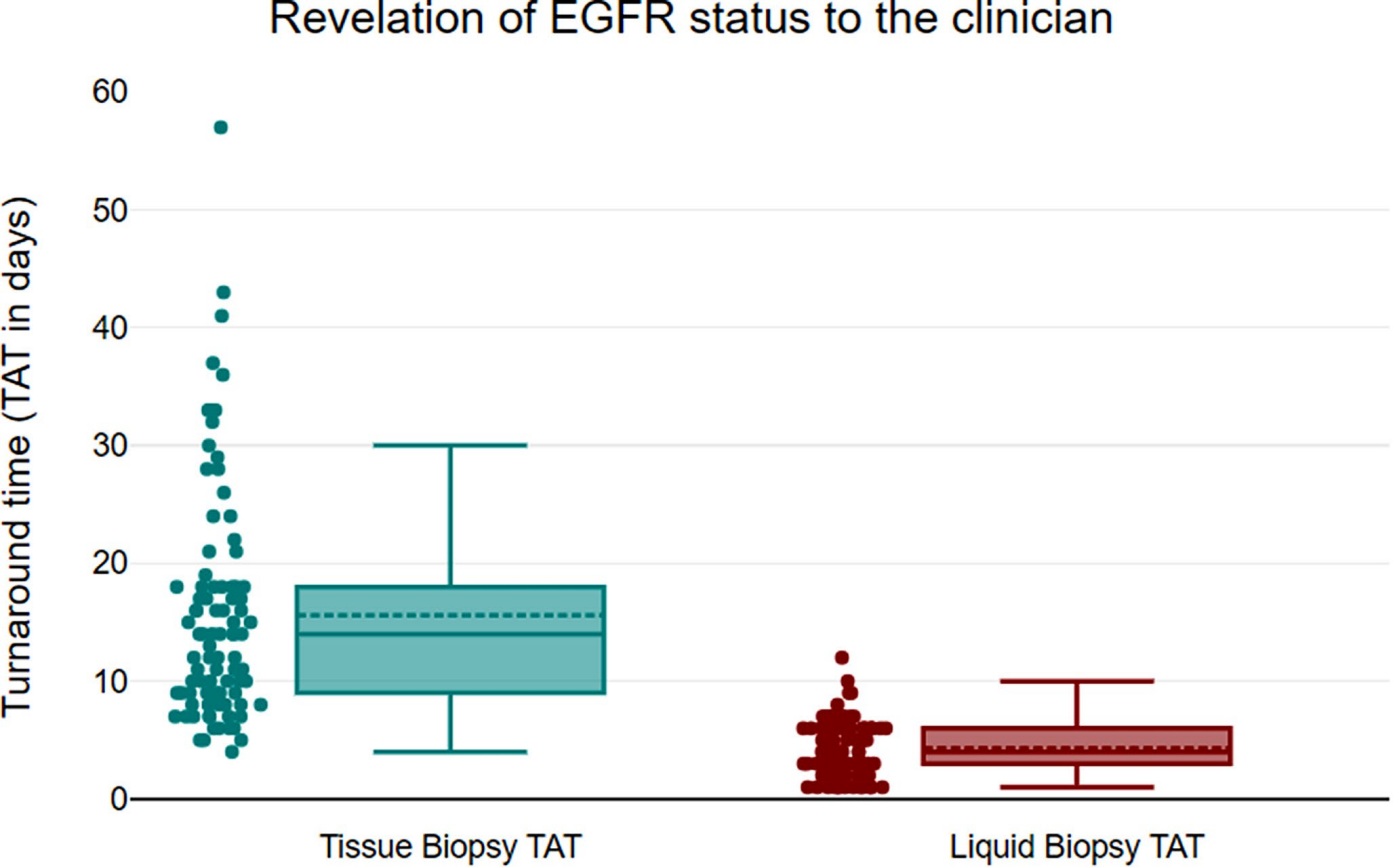
Turnaround time (TAT in days) for detection of *EGFR* mutation from matched tissue and plasma primarily tested using ARMS-PCR

All patients tested for *EGFR* mutation using liquid biopsy had shorter TAT (the time when the patient is presented to the clinic till the revelation of *EGFR* mutation status to the treating clinician) with a median TAT of 3 days (range 1–12 days) in comparison to the tissue *EGFR* testing with a median TAT of 12 days (range 7–57 days) with a significant p-value of < 0.05 (Fig. 3). There was no discordance in the target hotspot between matched plasma and tissue. The study showed that cfDNA testing for *EGFR* mutation detection using ddPCR had 82.8% sensitivity, 100% specificity, 100% positive predictive value (PPV) and 88.5% negative predictive value (NPV).

### Clinical features of *EGFR* mutant and *EGFR* wild type patients

Demographic details of *EGFR* mutated and *EGFR* wild type patients (Table 2) showed overall male predominance (male to female ratio of 1.22:1) of treatment naïve patients tested for *EGFR* mutations. However, it was found that females with *EGFR* mutation were more prevalent than males (*p* = 0.01). The median age of overall cohort was 55 years (range 28–84 years). Smoking history was available for 220/236 (93.2%) patients of which frequency of non-smoker was similar in both *EGFR* mutant and *EGFR* wild type group. However, the proportion of non-smokers in the *EGFR* mutant group was significantly higher than in the *EGFR* wild type group (*p* = 0.001).

**Table 2:** Patient demographic with clinicopathological details

Parameter	Patients with mutant EGFR	Patients with wild type EGFR	*p*-value
**Median Age (range) in years**	52 years (28–84 years)	57 years (28–82 years)	0.15
**Gender**	Total (*N* = 104)	Total (*N* = 132)	
Male	48 (46.2%)	82 (62.1%)	
Female	56 (53.8%)	50 (37.9%)	
Male: Female ratio	0.85:1	1.64:1	0.01
**Smoking history**			
Non-smoker	65	68	0.001
Smoker	18	51	
Tobacco	11	7	
**Diagnosis**			
NSCLC adenocarcinoma	94	116	-
NSCLC squamous cell carcinoma	-	2	
NSCLC-NOS	2	3	
NSCLC adenosquamous	-	6	
NSCLC (undifferentiated carcinoma)	4	-	
Poorly differentiated carcinoma	3	5	
Combined NSCLC and SCLC	1	-	

NSCLC = non-small cell lung carcinoma, SCLC = small cell lung carcinoma, NOS = not otherwise specified, EGFR = Epidermal growth factor receptor, N = numbers

### Treatment outcomes and survival of *EGFR* mutated patients

**Fig. 4:**
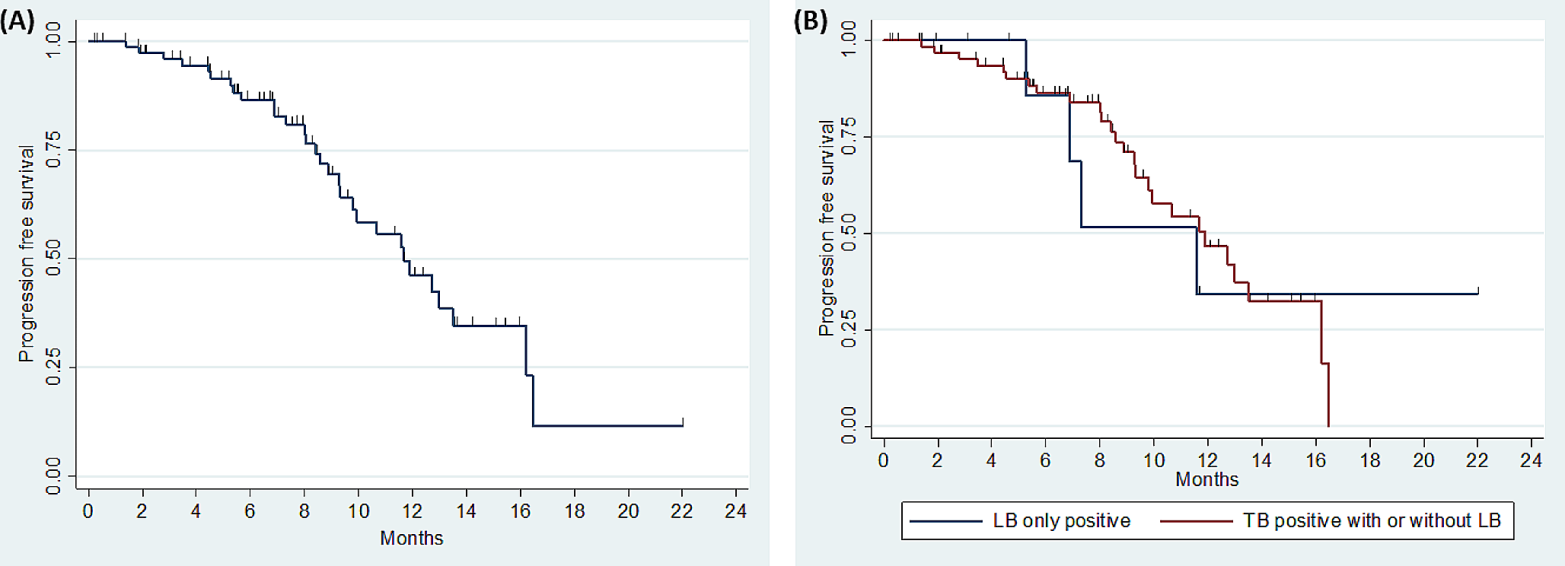
Kaplan-Meier survival analysis showing, **A**) Progression free survival (PFS) in all patients treated with EGFR TKI; **B**) Progression free survival (PFS) comparison of patients with *EGFR* status detected from liquid biopsy only and tissue biopsy with or without liquid biopsy (LB = liquid biopsy, TB = tissue biopsy)

Out of 104 *EGFR* mutant patients, treatment details of 96 (92.3%) patients were available (Supplementary Table 1). Among these, 85/96 (88.5%) patients received EGFR TKIs of which 66 (77.6%), 10 (11.8%), 3 (3.5%) and 6 (7.1%) were treated with gefitinib, erlotinib, afatinib and osimertinib, respectively. In the remaining 96 cases, eight (8.34%) patients received combination of EGFR TKI (gefitinib) and chemotherapy while three (3.1%) received chemotherapy only. Kaplan-Meier survival analysis was performed based on *EGFR* status detected by liquid biopsy only and by tissue biopsy with or without liquid biopsy. After the median follow-up of 12.6 months, progression-free survival (PFS) of all patients undergoing EGFR TKI therapy was found to be 11.67 months (95% CI 9.34–16.24; Fig. 4A). Those patients treated only on the basis of liquid biopsy *EGFR* status had similar PFS (11.56 months 95% CI 5.26-NR) when compared with those where *EGFR* status was detected by tissue biopsy with or without liquid biopsy (median PFS of 11.9 months 95% CI 9.34–16.23) (log rank *p* = 0.94) (Fig. 4B).

At disease progression till the last date of follow-up, repeat liquid biopsy with or without tissue biopsy could be performed in 17 of 96 cases (17.7%). (Supplementary Table 1). Repeat tissue biopsy could be performed in 8/96 (8.3%) patients with paired liquid biopsy in six patients. Overall, 19 patients were available with repeat liquid or tissue biopsy and tested for presence of T790M resistance mutation or histological transformation. Among these, 4 patients showed presence of *EGFR* T790M resistance mutation by both, ARMS-PCR and ddPCR. Additionally, 2 patients showed small cell transformation (SCT) and 13 showed either only the founder mutation or ctDNA cleared for founder mutation.

## Discussion

In recent years, treatment decisions of advanced stage unresectable NSCLC patients are mostly based on personalized medicine advancements. In advanced stage lung cancer, frontline liquid biopsy testing is recommended for *EGFR* mutation detection when tumor tissue is insufficient (Paweletz et al. 2016; Lindeman et al. 2018; Rolfo et al. 2021; Satapathy & Singh et al. 2021), while it was strongly recommended for detection in TKI resistance settings (Satapathy & Singh et al. 2021; Silveira et al. 2021; Filipits et al. 2023). The incidence of *EGFR* mutations in advanced stage NSCLC varies among different ethnicities, of which highest prevalence is observed among Asians (Benbrahim et al. 2018; Melosky et al. 2022; Hofman et al. 2023). Due to procedural and technical advantages of liquid biopsy, it has been widely accepted as an alternative to tumor tissue genotyping for detecting *EGFR* mutations in NSCLC (Paweletz et al. 2016; Leighl et al. 2019; Rolfo et al. 2021; Raez et al. 2023). Besides the limited availability of tissue biopsy for tumor genotyping, turnaround time is a critical factor for lung cancer patients with a heavy symptomatic disease burden. In such scenario, it is prudential to adopt testing strategies that help in quickly identifying patients eligible for targeted therapy by single-gene testing such as *EGFR* oncogenic driver mutations in comparison to the more comprehensive NGS based approaches. Furthermore, in regions of the world with high *EGFR* mutation rates, the initial molecular evaluation often involves limited PCR analysis for detecting *EGFR* mutations (Rolfo et al. 2021).

We performed single gene *EGFR* mutation testing by two methods, using real-time polymerase chain reaction based followed by ddPCR. Real-time PCR based methods have been widely used due to their cost-effectiveness and reliable results (Hofman et al. 2023). In our study, *EGFR* mutation detection using ARMS-PCR method had a lesser sensitivity of 66.34% (69/104) as shown by other trials in comparison to ddPCR (Li et al. 2019; Satapathy & Singh et al. 2021; Douillard et al. 2014; Hsiue et al. 2016; Suryavanshi et al. 2018; Satapathy & Singh et al. 2021). Real-time PCR based assays have been widely used for detecting *EGFR* mutations in tumors and has shown limited clinical sensitivity when it comes to detecting *EGFR* mutations in liquid biopsy samples. We observed false-negative plasma results using ARMS-PCR in 35 cases with *EGFR* mutations. This further highlights the challenge of detecting *EGFR* mutations in liquid biopsy samples due to the low quantity of cfDNA. Liquid biopsy has proven to be an invaluable tool in identifying *EGFR* mutations in NSCLC patients. Through the utilization of highly sensitive ddPCR and NGS techniques, previous studies have demonstrated the remarkable sensitivity and specificity of this approach (Pawaletz et al. 2016; Wei et al. 2019; Soria-Comes et al. 2020; Satapathy & Singh et al. 2021).

However, various factors have been identified limiting the clinical sensitivity and false-negative results with plasma mutation analysis (Trigg et al. 2018; Markus et al. 2018; Aldae et al. 2020; Song et al. 2022). Plasma contains tumour-derived circulating tumor DNA (ctDNA), with the proportion of ctDNA in the bloodstream being influenced by the release from tumor cells undergoing apoptosis and necrosis. Aldae et al. have demonstrated a significantly lower shedding of ctDNA between NSCLC patients with central nervous system (CNS) metastases during disease progression and those without any CNS involvement. Various pre-analytical factors impact the quantity of cfDNA in the blood (Trigg et al. 2018; Markus et al. 2018). Additionally, patient related factors frequently contribute to the effectiveness of mutation detection, particularly in cases where there is a minimal presence of mutant DNA (Zhu et al. 2015).

The utilization of ddPCR assays to detect the low limit of detection enhances its suitability as a more sensitive approach for identifying mutations in liquid biopsy samples. ddPCR assays have demonstrated a sensitivity in detecting *EGFR* mutation as low as 0.04%, with the detection limit depending on the sample DNA input and the ratio of mutant copies to wild-type DNA template (Zhu et al. 2015). We experienced failure in detecting one-third (35 out of 104 cases) of total *EGFR* positive cases using ARMS-PCR. Of these 35 cases, 2 cases were positive for exon 20 insertions and exon 18 G719x mutation which were not technically feasible using ddPCR. In the remaining 33 cases, ddPCR successfully detected mutations in approximately 51.5% (17/33) of the cases. Out of these, 12 cases were positive for exon 21 L858R mutation, and ddPCR was able to detect 9 out of these 12 cases. However, ddPCR identified only 8 out of 22 false- negative cases with exon 19 deletions.

We have observed cases that were initially false-negative but later tested positive in plasma using ddPCR, with mutant DNA fractions ranges from 0.1 to 0.9%. Such cases with very low tumor fraction may be attributed to cfDNA contamination by non-tumor DNA reducing the fraction of tumor derived DNA and thereby, false negative plasma results with less sensitive ARMS-PCR method. However, a significant number of false negative plasma samples (14 out of 22 cases) with exon 19 deletions went undetected using ddPCR. This could also be attributed to the utilization of the E746_A750del mutation assay in ddPCR, which is the common subtype for del 19, (Rossi et al. 2019; Zhao et al. 2020). It remains unclear whether other uncommon subtypes of exon 19 deletion mutation differ from the common one in terms of tumor DNA shedding. In a recently published study, we observed a case of exon 19 deletion with uncommon *EGFR* subtype (Leu747-thr751delinsGln) with a mutant fraction as high as 87.5%. Surprisingly, this uncommon *EGFR* mutation subtype went undetected by the less sensitive PCR-based assay but was successfully identified through NGS (Thakur & Rathor et al. 2024). Similarly, another study identified unusual L858R mutation identified using NGS in liquid-based cytology indicating that NGS based methods are superior than PCR-based methods in detecting more mutation sites within a target region (Wu et al. 2020). Furthermore, within a group of patients who tested negative on liquid biopsy results, we identified 2.8% (3/104) of cases with exon 20 insertions, with only one of these cases showing a negative result on liquid biopsy. EGFR Exon 20 insertions are heterogenous short in-frame insertions which are the third most frequent *EGFR* mutations in NSCLC (Burnett et al. 2021). While traditionally these mutations are associated with a poorer prognosis compared to classical *EGFR* mutations (Chouaid et al. 2021), the recent approval of selective inhibitors has sparked renewed interest in studying and targeting these specific mutations (Passaro et al. 2022). Detection of EGFR exon 20 insertions has been earlier limited to the use of multiplex-based PCR kits, which have shown significant false-negative results when compared to NGS (Shen et al. 2022; Rolfo et al. 2023).

The present study suggests that the plasma first approach can overcome a major implementation barrier for personalized medicine i.e. long waiting time of invasive tissue biomarker results (Aggarwal et al. 2019; Hofman et al. 2023). The estimated TAT in the clinical guidelines for *EGFR* testing using tissue biopsy is 7–10 days (Hofman et al. 2023) however significantly shorter TAT can be achieved using the ‘plasma first’ approach in comparison to tissue biopsy (median TAT of 3 vs. 12 days, respectively; p=<<0.05). We have shown how implementing ‘plasma first’ approach is linked to a significant improvement in the TAT as short as 1 day to reveal *EGFR* status to the clinician. Similar studies were performed using NGS based testing that demonstrated dispensability of liquid biopsy in determining front-line therapy decision with shorter TAT and greater test success rate in comparison to tissue biopsy (Aggarwal et al. 2019; Cui et al. 2022; Raez et al. 2023; García-Pardo et al. 2023; Russo et al. 2024). The challenge lies in obtaining matched tissue biopsy samples for patients with poor clinical conditions or when biopsies are not feasible. Consequently, this has led to a biased increase of 44.06% in the *EGFR* mutations.

Additionaly, we evaluated progression-free survival (PFS) of patients who received EGFR TKI therapy. The PFS did not significantly differ between patients treated based on liquid biopsy alone versus those treated based on tissue biopsy with or without liquid biopsy (median PFS of 11.56 vs. 11.9 months, respectively; *p* = 0.94). The observed PFS with EGFR TKIs was similar as reported in various studies and treatment decision based on liquid biopsy do not affect clinical outcomes (Huang et al. 2021; Lu et al. 2023). The ‘plasma first’ approach allowed clinician to treat patients with positive cfDNA results for *EGFR* single oncogene test. Although tumor tissue is the ‘gold standard’ for tumor genotyping, it still remains undergenotyped in many patients (Smolle et al. 2021). In the present study, tissue *EGFR* status was either unknown or not sufficient for *EGFR* molecular testing in twelve cases however, in these patients liquid biopsy was the only tool for predicting *EGFR* status. The study showcased the PFS of patients who underwent treatment solely based on their *EGFR* status, utilizing a single-gene testing approach. We also observed that nearly one-third of the *EGFR*-positive patients who received EGFR TKI therapy experienced a PFS duration of less than 5 months. Recent studies have shed light on the correlation between co-mutations and unfavorable outcomes, as well as the underlying mechanism that promotes resistance in *EGFR*-mutant lung adenocarcinoma (Vokes et al. 2022; Liu et al. 2022).

In addition, the NSCLC subtype is dynamically evolving with current recommendations suggesting testing with a multigene NGS based approach (Mosele et al. 2020; Ettinger et al. 2022). The primary limitation of the current study lies in exclusive testing of the *EGFR* gene through liquid biopsy, instead of conducting comprehensive multi-gene testing that includes comutations. Studies using NGS on liquid biopsy in metastatic advanced stage have shown the potential of using liquid biopsy as a standard of care to complement tissue genotyping (Paweletz et al. 2016; Aggarwal et al. 2019; Leighl et al. 2019). Some recent NGS based studies evaluated the potential of using plasma NGS approach in subjects with suspected lung cancer prior to obtaining tissue biopsy (Cui et al. 2022; Raez et al. 2023; García-Pardo et al. 2023; Russo et al. 2024). García-Pardo et al. and Cui et al. demonstrated similar median turnaround time (TAT) of approximately one week for plasma-based NGS compared to tissue diagnosis, which had a median TAT of around three weeks in advanced nonsquamous NSCLC. We are conducting an investigative study utilizing NGS based approach to evaluate the potential of using liquid biopsy in the management of treating patients with concomitant mutations. The current project served to evaluate the feasibility of integrating liquid biopsy into standard patient care for the most prevalent predictive biomarker, *EGFR*.

## Conclusions

Liquid biopsy is non-invasive, offers high specificity and an efficiently quick testing method compared to tissue biopsy sampling, which may not always be feasible or sufficient for molecular testing. It can serve as an alternative for biomarker evaluation during initial diagnosis to detect *EGFR* mutations in advanced NSCLC. The turnaround time (TAT) for *EGFR* molecular analysis using liquid biopsy is significantly faster than tissue biopsy, thereby resulting in reduced delays in treatment. The present study indicates that survival outcomes are similar between liquid biopsy and tissue biopsy, suggesting that liquid biopsy is a promising modality for early detection of *EGFR* mutations in advanced NSCLC, especially in parts of globe where *EGFR* mutation rate is high.

### Electronic supplementary material

Below is the link to the electronic supplementary material.

Supplementary Material 1

Supplementary Material 2

## Data Availability

The datasets generated during and/or analysed during the current study are available from the corresponding author on reasonable request.

## Declarations

### Presentation at a meeting

The work was presented as an e-poster at the World Conference on Lung Cancer (WCLC) 2022, organised by International Association for the Study of Lung Cancer (IASLC).
