# Supplementary material for: ‘Plasma first’ approach for detecting epidermal growth factor receptor mutation in advanced non-small cell lung carcinoma
Source: J Cancer Res Clin Oncol. 2024 Jul 27;150(7):371. doi: 10.1007/s00432-024-05828-w (PMC11283418; doi:10.1007/s00432-024-05828-w)
Supplement: Supplementary file 1 — Supplementary Material 1 [file 432_2024_5828_MOESM1_ESM.docx]

**Supplementary Information:**

**Supplementary Figure 1:** (A) Real-time PCR amplification plot showing case 1 with tumor tissue scant for molecular analysis but cfDNA found to be positive for EGFR exon 19 deletion; (B) showing amplification plot of a case 2 with compound mutation detected by ARMS-PCR (exon 21 L858R and exon 20 T790M) (C) cfDNA EGFR status showing exon 19 deletion droplets marked in green circle from sample shown in plot A confirmed using ddPCR; (D) cfDNA from a compound mutation case 51 tested positive for L858R using ddPCR and mutation positive droplets marked in red circle

**Supplementary Table 1:** Treatment details with EGFR mutation subtype and follow-up data of patients on disease progression


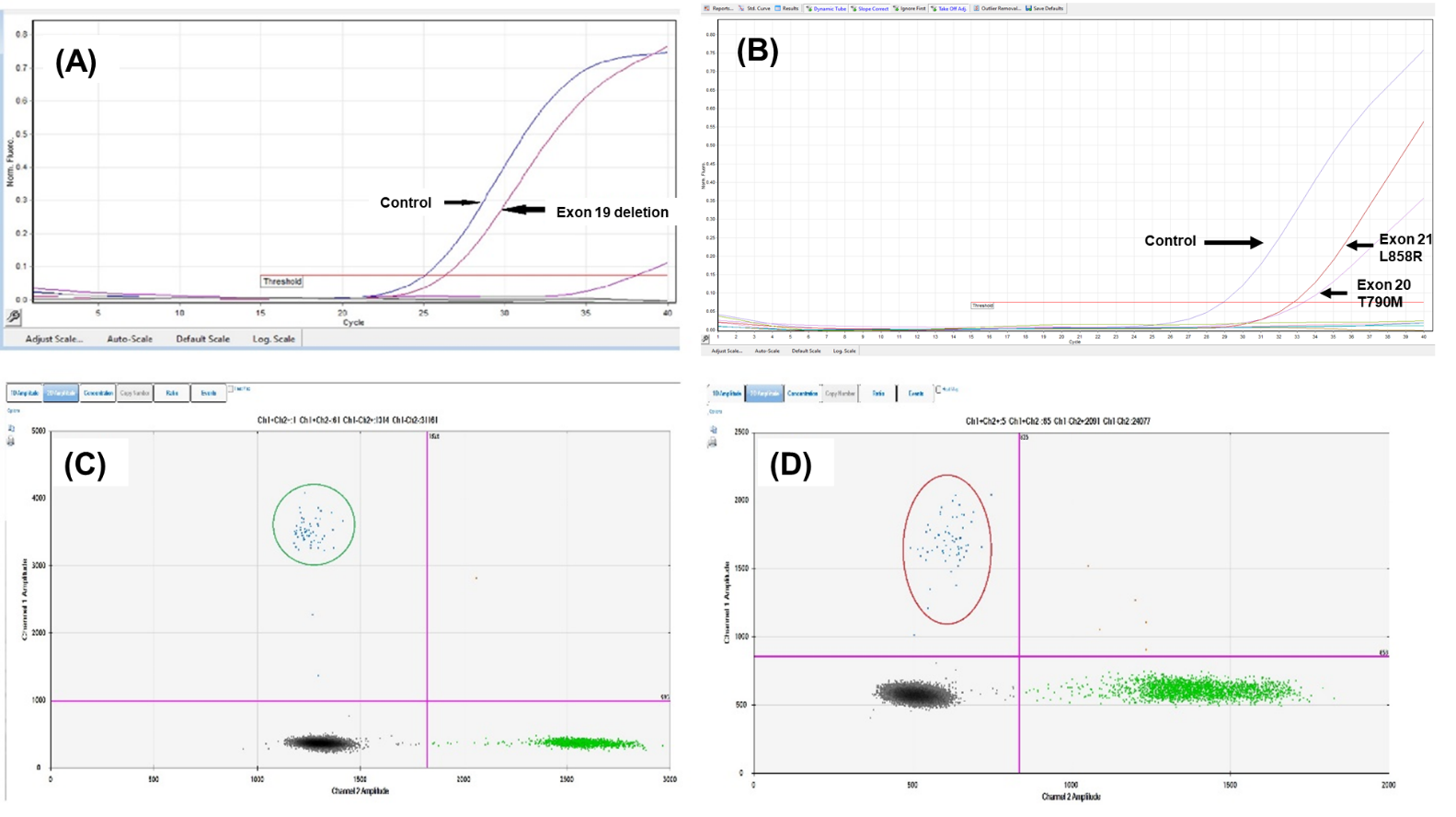


**Supplementary Figure 1:** (A) Real-time PCR amplification plot showing case 1 with tumor tissue scant for molecular analysis but cfDNA found to be positive for EGFR exon 19 deletion; (B) showing amplification plot of a case 2 with compound mutation detected by ARMS-PCR (exon 21 L858R and exon 20 T790M) (C) cfDNA EGFR status showing exon 19 deletion droplets marked in green circle from sample shown in plot A confirmed using ddPCR; (D) cfDNA from a compound mutation case 51 tested positive for L858R using ddPCR and mutation positive droplets marked in red circle

**Supplementary Table 1:** Treatment details with *EGFR* mutation subtype and follow-up data of patients on disease progression

| **Parameter** | **All** | **Single common** | **Single uncommon** | **Compound** |
| --- | --- | --- | --- | --- |
| **Treatment administered (N=104)**  EGFR TKI  Chemotherapy  Combination of EGFR TKI & chemotherapy  Treatment not started or lost to follow-up | 85 (81.7%)  3 (2.9%)  8 (7.7%)  8 (7.7%) | 83  1  7  5 | 2  2  -  1 | -  -  1  2 |
| **Disease Progression (N=96)**  Yes  No | 24 (25%)  72 (75%) | 23  68 | -  3 | 1  - |
| **Repeat biopsy on disease progression (N=24)**  Tissue Biopsy  Liquid Biopsy  Both Tissue and liquid biopsy  Not available | 2 (8.34%)  11 (45.84%)  6 (25%)  5 (20.84%) | 2  11  6  5 | -  -  -  - | -  -  -  - |
| **Status on disease progression (N=19)**  Founder mutation (+) and T790M positive  Founder mutation only  Small cell transformation (SCT) | 4 (21%)  13 (68.4%)  2 (10.6%) | 4  12  2 | -  -  - | -  1  - |
| EGFR= Epidermal growth factor receptor, TKI= tyrosine kinase inhibitors; N= numbers | | | | |
