# Supplementary figures and images for: ‘Plasma first’ approach for detecting epidermal growth factor receptor mutation in advanced non-small cell lung carcinoma
Source: J Cancer Res Clin Oncol. 2024 Jul 27;150(7):371. doi: 10.1007/s00432-024-05828-w (PMC11283418; doi:10.1007/s00432-024-05828-w)

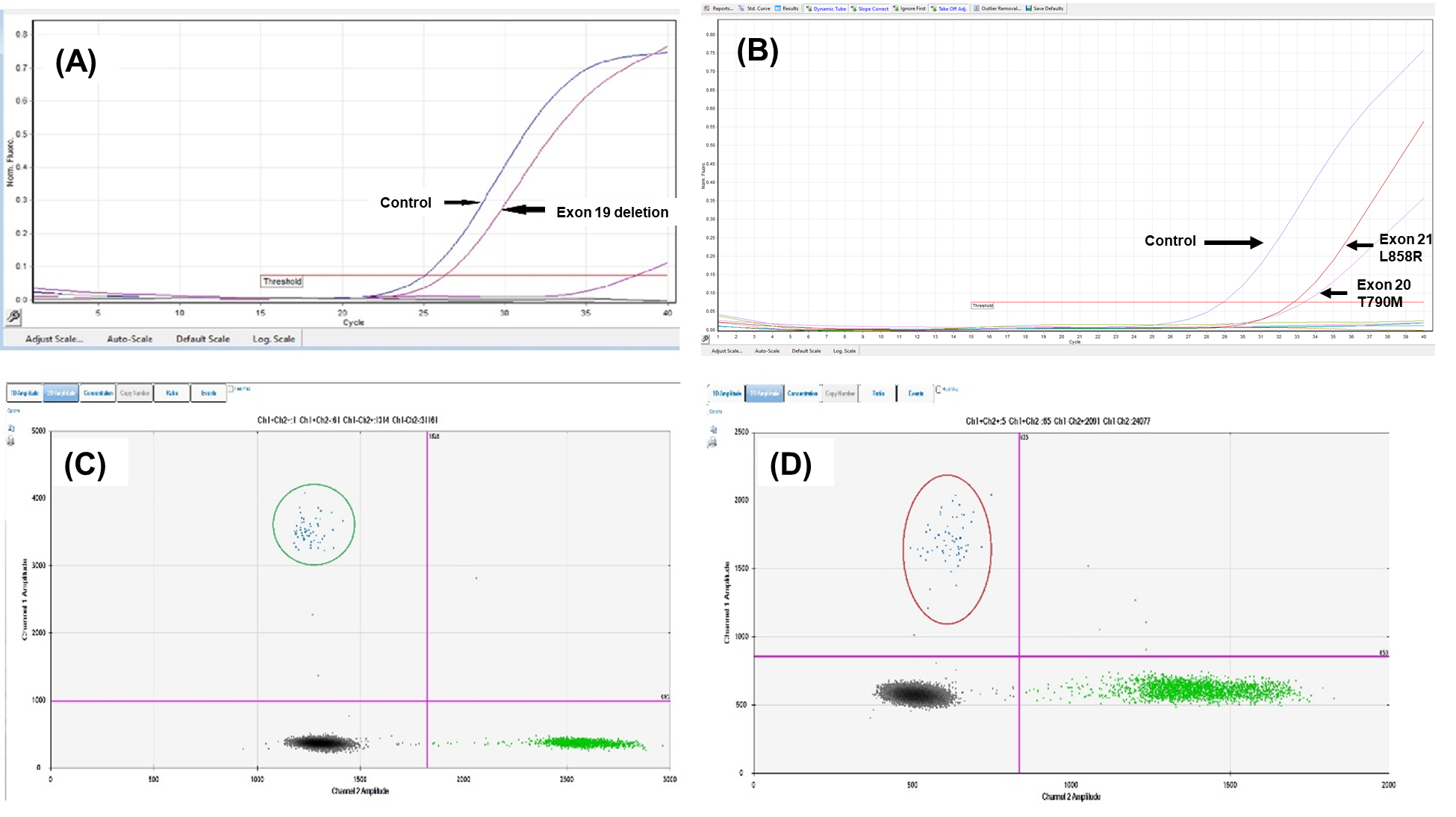

Supplement: Supplementary file 2 — Supplementary Material 2 [file 432_2024_5828_MOESM2_ESM.tif]
